# Embedding Trauma-Informed Strategies Within a Multitiered System of Supports: A Framework for Early Childhood Education

**DOI:** 10.3390/bs15121640

**Published:** 2025-11-28

**Authors:** Freddie Pastrana Rivera, Zachary C. LaBrot, Brittany Garza, Josselyn Telule, Lourdes Rodriguez, Abigail Ann Wilkinson

**Affiliations:** School of Psychology, University of Southern Mississippi, Hattiesburg, MS 39406, USA; freddie.pastranarivera@usm.edu (F.P.R.); brittany.garza@usm.edu (B.G.); josselyn.telule@usm.edu (J.T.); lourdes.rodriguez@usm.edu (L.R.); abigail.wilkinson@usm.edu (A.A.W.)

**Keywords:** trauma-informed, multitiered supports, MTSS, PBIS, early childhood, early intervention, program-wide positive behavior supports

## Abstract

Program-wide Positive Behavior Supports (PW-PBIS) is a data-driven multitiered model of service delivery in which young children receive increasingly intensive supports to address their social–emotional and behavioral needs. PW-PBIS implementation in early childhood education has consistently been found effective for preventing a variety of undesirable outcomes while also promoting young children’s social–emotional and behavioral functioning. However, the literature examining the PW-PBIS model has often overlooked the impact of early adversity and trauma as factors on young children’s outcomes. Emerging studies have begun proposing a trauma-informed lens be implemented in early childhood education; however, the PW-PBIS model lacks clear guidelines on how to adapt interventions and supports at each tier to be trauma-informed. Therefore, using a trauma-informed lens, this paper aims to offer evidence-guided approaches to adapt the PW-PBIS framework. Practical trauma-informed adaptations of specific interventions and supports at each PW-PBIS tier will be described. Further, a data-based case example will be presented and discussed to illustrate the utility of a trauma-informed PW-PBIS model. The goals of this paper are to advise practitioners on how PW-PBIS can be adapted to be trauma-informed while also serving as a call to action for researchers to empirically investigate this model.

## 1. Introduction

### 1.1. Program-Wide Positive Behavior Interventions and Supports in Early Childhood Education

Program-Wide Positive Behavior Interventions and Supports (PW-PBIS; Hemmeter et al., 2007) is a model used to identify, prevent, and intervene with children in early childhood education settings at risk for the development of social–emotional and behavioral difficulties. PW-PBIS is an adaptation of School-Wide Positive Behavior Interventions and Supports (SW-PBIS), which targets students in kindergarten through 12th grade (Sugai & Horner, 2006). The overarching goal of PW-PBIS is to ultimately prevent social–emotional and behavioral challenges by systematically targeting children’s social–emotional and behavioral competencies. Moreover, given the high prevalence of social–emotional and behavioral difficulties exhibited by children in early education (Snell et al., 2012; Yoder & Williford, 2019), PW-PBIS is designed for efficiency by only providing certain levels of support to those children who warrant the need. These goals are accomplished by consistently implementing five essential components of PW-PBIS, with their primary focus being the prevention of problems (Fox et al., 2010; Hemmeter et al., 2007). First, PW-PBIS involves regular universal screening of social–emotional and behavioral development to identify children at risk for developing problems; this allows for the early identification, intervention, and prevention of worsening problems. Beyond initial universal screening, a second critical feature of PW-PBIS is continuous progress monitoring of a child’s progress, or lack thereof, to determine if supports should be faded or intensified. Relatedly, a third feature is data-based decision-making, such that decisions about whether to decrease or increase supports are made based on universal screening and continuous progress monitoring data. A fourth feature requires that all practices be implemented with fidelity and that data be collected on implementation fidelity. Finally, perhaps the most iconic feature of PW-PBIS is that evidence-based interventions and supports are implemented within a continuum of three tiers that increase with intensity and focus as children fail to respond to less intensive tiers (i.e., Tiers 1, 2, and 3; Fox et al., 2010; Hemmeter et al., 2007).

Tier 1 consists of universal screening and preventative strategies to create safe and supportive relationships and environments, such as teaching behavioral expectations, acknowledging children for following the expectations, and establishing predictable routines (Fox et al., 2010; Hemmeter et al., 2007). These are commonly achieved through teacher implementation of universally delivered practices such as behavior-specific praise (BSP; Royer et al., 2019), effective instruction (DeFouw et al., 2025; LaBrot et al., 2024; Johnson et al., 2024), precorrections (R. P. Ennis et al., 2017), and positive feedback and encouragement during transitions (LaBrot et al., in press), to name a few. Behavior incident reports serve as data to assess the frequency and types of challenging behaviors that occur in the context of Tier 1 and are used to inform those children who require Tier 2 supports (Hemmeter et al., 2007).

Tier 2 consists of more targeted supports that focus on explicit instruction of social–emotional and behavioral skills (Fox et al., 2010; Hemmeter et al., 2007). This often involves tailoring interventions and supports to specifically focus on the social–emotional and behavioral difficulties exhibited by selected children, as opposed to broad universal supports. For example, this may involve providing prompts and feedback on specific social–emotional skills or behaviors, implementing social skills interventions in small groups, or developing relatively low-intensive targeted interventions and supports unique to a child. Specific Tier 2 supports implemented within a PW-PBIS model have included, but are not necessarily limited to, check-in/check-out (e.g., LaBrot et al., 2016; LeBel et al., 2013), class-wide group contingencies (e.g., Pasqua et al., 2021; Pokorski et al., 2017), training teachers in specific classroom management skills (e.g., DeFouw et al., 2025; Gomez et al., 2021; Johnson et al., 2024; LaBrot et al., 2024), and small group interventions (e.g., Daunic et al., 2013; Stanton-Chapman et al., 2014). As with Tier 1, progress monitor data and behavior incident reports are collected to determine whether children are making progress and can be transitioned back down to Tier 1 supports or transitioned to Tier 3.

Tier 3 supports are informed by functional assessment of challenging behaviors and environmental variables that trigger and maintain them (Fox et al., 2010). As such, these supports are highly individualized and much more intensive than supports at Tiers 1 and 2 (LeGray et al., 2013; LaBrot et al., 2018; von Schulz et al., 2018). After a period of progress monitoring, children may be transitioned back to Tier 2 and eventually Tier 1. Supports delivered at Tiers 2 and 3 are both cumulative, such that children receive both Tiers 1 and 2 supports at Tier 2, and children receive all three levels of support at Tier 3.

### 1.2. Research Supporting PW-PBIS

Overall, research demonstrates that PW-PBIS is effective for improving young children’s social–emotional and behavioral outcomes. Specifically, improvements in language skills (e.g., receptive and expressive language), early learning outcomes (e.g., phonological awareness), social–emotional skills, class engagement, and challenging behaviors have been reported. Additionally, research indicates that PW-PBIS helps teachers implement evidence-based interventions and supports with fidelity (Shepley & Grisham-Brown, 2019). Despite these positive outcomes, there has been less focus on PW-PBIS’s role in addressing challenges in young children who have experienced adversity and trauma.

In fact, early childhood professionals report needing training and support in understanding childhood trauma and how this translates into classroom management practices (Chudzik et al., 2024). As recognition grows that trauma exposure can negatively impact children’s adjustment, researchers have developed frameworks for and begun evaluating the effects of school-based trauma-informed interventions (Maynard et al., 2019; Panlilio, 2019; Thomas et al., 2019; Vachhani et al., 2025; Wiest-Stevenson & Lee, 2016). Though promising, widespread implementation is still lacking (cf. Hoover et al., 2018), as is the integration of trauma-informed programming within existing multitiered support systems (e.g., PW-PBIS). While recent efforts to integrate these are underway (Eber et al., 2020; Riggs & Landrum, 2023), the research evaluating trauma-informed multitiered supports has focused on elementary, middle, and high schools rather than early childhood (Berger, 2019). As such, this paper aims to review the experiences of preschool-age children exposed to adversity and trauma and describe a model of PW-PBIS in early childhood settings that integrates trauma-informed supports at each tier. Moreover, we provide an illustrative case example informed by case study methods to demonstrate the application of this model.

## 2. Adversity and Trauma in Early Childhood: Implications for PW-PBIS

Children are often exposed to adversity and trauma, which can negatively impact their health and well-being. Adverse childhood experiences (ACEs) occur before age 18 and include abuse (e.g., physical, sexual, psychological); witnessing intimate partner violence; caregiver loss, incarceration, mental illness, substance misuse, or suicidality; and other adverse conditions (e.g., housing instability, food insecurity) (Cain et al., 2022; Merrick, 2019). In the U.S., an estimated two-thirds of children report at least one ACE by age 16, with one in seven experiencing abuse or neglect every year (SAMHSA, 2014). While less work has focused on young children, studies report a range of 12–70% of early childhood samples endorsing three or more distinct ACEs (Liming & Grube, 2018). Given the prevalence of ACEs, it is necessary to understand their impact on young children’s adjustment.

Ample evidence links early ACEs to children’s maladjustment risk. For example, Jimenez et al. (2016) found that exposure to 3+ ACEs in early childhood was associated with below-average academic skills (e.g., language, literacy, math) and increased problems (e.g., attention, social, aggression) in kindergarten. Studies find a dose–response effect of ACEs on functioning, with cumulative exposure predicting higher risk. From a national study (children ages 1 to 5), 24% of those with no ACEs showed risk for developmental, social, or behavioral delays, yet over 42% of those reporting 4+ ACEs endorsed delays (Cprek et al., 2020). ACEs have been negatively associated with prosocial behavior and positively with long-term risk, including externalizing and internalizing symptoms (Gautam et al., 2024; X. Wang et al., 2022), behavior problems in middle childhood and adolescence (Choi et al., 2019), psychiatric (e.g., depressive, anxiety) and substance use disorders in adults (Daníelsdóttir et al., 2024), and even reduced functioning in later adulthood (Haczkewicz et al., 2024).

In addition to ACEs, children risk exposure to *potentially traumatic events* (PTEs), events or circumstances resulting in physical, emotional, or life-threatening harm (SAMHSA, 2014). PTEs include witnessing and experiencing neglect and abuse (e.g., sexual, physical); interpersonal (e.g., intimate partner, assault), community, and mass violence (e.g., war, terrorism); sudden and violent loss; serious accidents and injuries; and natural (e.g., tornado, earthquake) and human-made disasters. Prevalence rates in the U.S. are striking; over 70% of adults report at least one lifetime PTE (Benjet et al., 2016), and estimates suggest 14–43% of children risk significant exposure (The National Child Traumatic Stress Network, 2025). Early childhood trauma typically refers to traumatic events occurring before age 6 (Clarkson Freeman, 2014). Though preschool-aged children are susceptible to a wide range of PTEs (Bartlett & Smith, 2019; The National Child Traumatic Stress Network, 2025), prevalence rates for this age are difficult to ascertain due to underreporting by adults and misunderstanding of events by children (Saunders & Adams, 2014). While rates remain unclear, estimates suggest 26–70% of young children in the U.S. may experience a PTE before kindergarten (Bartlett, 2021; Briggs-Gowan et al., 2010). Such estimates are concerning, especially for children who might be particularly susceptible to the harmful consequences of trauma during critical developmental periods.

Not all children exposed to adversity evidence enduring consequences (Copeland et al., 2007; Galatzer-Levy et al., 2018), with many exhibiting recovery and resilient trajectories (Andersen et al., 2014; Lai et al., 2017). Nevertheless, research still points to a significant association between early trauma and negative outcomes (Cook et al., 2005; Downey & Crummy, 2022; Dye, 2018; McLaughlin et al., 2013), especially for children with risk vulnerabilities and those exposed to multiple adverse events. Posttraumatic reactions may include re-experiencing the event (e.g., nightmares, intrusive memories), avoidance of stimuli or cues that trigger memories of the event, changes in thoughts (e.g., negative beliefs about the world or self) or mood (e.g., sadness, anger, feeling numb), and changes in arousal or reactivity (e.g., easily startled, hypervigilant, disturbed sleep) (Alisic et al., 2008). Though many might experience transitory or brief post-trauma reactions, a smaller proportion exhibit clinical concerns.

From PreK through 12th grade, evidence suggests early adversity and trauma may increase risk for impairment in school success and educational outcomes (Mullins & Panlilio, 2021; Panlilio et al., 2018; Schatz et al., 2008; Schultz et al., 2009). Early trauma enhances risk for challenges with behavior and emotion regulation (Ferrara et al., 2023), impulse control and executive functions, and externalizing disorders (e.g., ADHD, ODD) (Tobin & Oldfield, 2016). Trauma can impact children’s capacity to self-regulate, follow instructions, and interact appropriately with peers and adults at school. They may show difficulty attending and focusing on lessons and learning, integrating, and applying concepts. Trauma-exposed children have historically been more likely to use academic supports, exhibit lower academic achievement, and show higher rates of grade repetition and dropout (Tobin & Oldfield, 2016). Concerningly, they may also evidence risk for impaired cognitive and intellectual functioning (Hart & Rubia, 2012; Pechtel & Pizzagalli, 2011).

### Advocating for a Trauma-Informed Lens in Early Childhood PW-PBIS

Given the myriad of possible adverse outcomes in young children exposed to adversity and trauma, it is imperative to investigate mechanisms that could buffer against potentially negative trajectories. Consequently, school-based professionals and early childhood educators should continue making efforts to mitigate children’s risk for trauma exposure and trauma-related sequelae. School professionals working closely with children typically possess a broad, foundational knowledge of trauma; however, they report lacking a specific understanding of trauma and its effects (Ferrara et al., 2023). With proper professional development and training, early childhood providers may be in a unique position to promote resilience and post-trauma recovery and prevent negative socioemotional and behavioral outcomes and long-term impairment in young children exposed to trauma. One potential avenue to address this gap may be to integrate trauma-informed practices into early childhood education settings.

Trauma-informed care is a framework that acknowledges the prevalence and impact of trauma, identifies trauma-related signs and symptoms in individuals, integrates trauma-specific knowledge into policies, procedures, and practices, and actively seeks to prevent re-traumatization (Center for Health Care Strategies, 2022; Sun et al., 2024b). Rather than functioning as a stand-alone intervention, trauma-informed care serves as a guiding perspective that can be used to adapt and enhance existing systems of support to better meet the needs of children affected by trauma. The PW-PBIS framework lends itself to a trauma-informed lens given its emphasis on the identification, prevention, and intervention of social–emotional and behavioral difficulties—symptoms frequently exhibited in children exposed to trauma. Viewing PW-PBIS through a trauma-informed lens may strengthen its relevance, responsiveness, and overall impact within early childhood settings.

Integrating a trauma-informed lens into the existing PW-PBIS framework may encourage educators to ask, “What are this child’s lived experiences?” and “Are their behaviors related to those experiences?” rather than “What’s wrong with this child?” and “Why won’t this child just do what I’m telling them?” This shift in perspective reframes challenging behaviors as potential indicators of underlying needs and moves the emphasis from enforcing compliance to prioritizing understanding, emotional well-being, support, and safety. Applying a trauma-informed lens to PW-PBIS also underscores the importance of cultivating secure, trusting relationships between children and educators. For many children, teachers serve as a primary source of care and social support (Alisic et al., 2012; Farmer et al., 2011). This role becomes particularly critical when considering children who have been exposed to trauma. Within a trauma-informed PW-PBIS framework, educators would prioritize emotional safety and the development of regulation skills, creating an environment that supports children’s capacity to participate fully and meaningfully in learning. By providing educators with trauma-informed strategies to proactively support behavior and emotion regulation, this approach has the potential to promote positive long-term outcomes, including improved social–emotional competence and readiness to learn.

## 3. Integrating Trauma-Informed Practices in Each Tier of Early Childhood PW-PBIS

**Figure 1:**
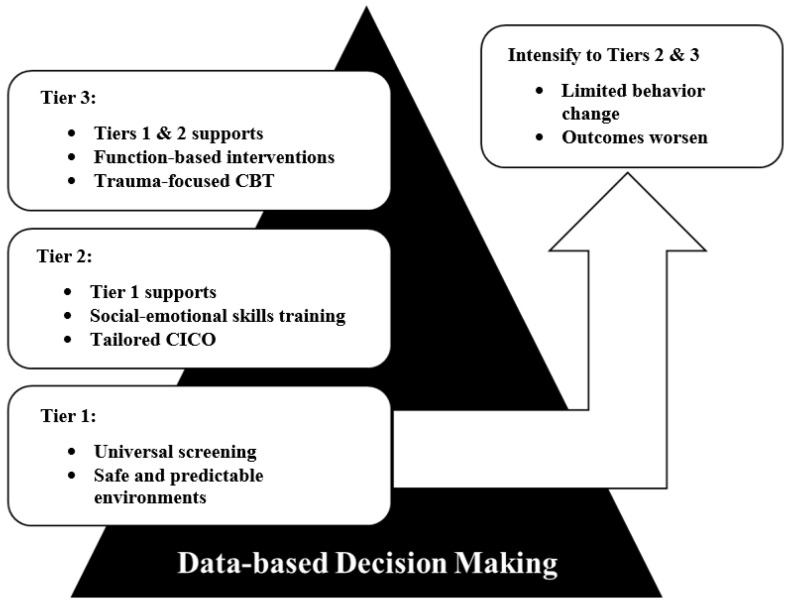
Diagram of Trauma-informed PW-PBIS Model.

A defining feature of PW-PBIS is its tiered framework, in which supports are delivered with increasing intensity and specificity to children not responding to less intensive levels of intervention (Fox et al., 2010; Hemmeter et al., 2007). Integrating trauma-informed practices within this structure enables each tier to address not only skill acquisition but also the distinct relational, emotional, and environmental needs of children who have experienced trauma and adversity. While emerging scholars have made the call for trauma-informed PW-PBIS (e.g., Riggs & Landrum, 2023), lacking are recommendations for doing so in early childhood contexts. The following sections outline sample ideas of how each tier of PW-PBIS could be adapted to integrate trauma-sensitive strategies while maintaining fidelity to the core principles of the model in early childhood educational settings. See Figure 1 for a diagram of the proposed model of integrating trauma-informed practices into PW-PBIS.

### 3.1. Tier 1

Tier 1 of PW-PBIS is designed to promote safe, predictable, and nurturing environments for all children. In a traditional PW-PBIS framework, all students receive universal screening and supports, irrespective of life experiences. However, early childhood education providers report lacking sufficient education, training, and supervision related to early childhood trauma (Bartlett & Smith, 2019; Hunter et al., 2021). Teachers also report needing more training on effective approaches to manage the classroom and support students before, during, and after emergent crises and PTEs (Machado & Anderson, 2023). Educators may benefit from universal professional development on adversity and trauma that could interfere with children’s social–emotional and academic functioning. This could include learning about trauma prevalence, common child reactions, short- and long-term consequences, and potential risk and protective factors, as well as practices teachers could implement in their classrooms.

At Tier 1, continuing to foster a predictable learning environment for all learners is essential. Children exposed to trauma require structure, consistent routines, and clear expectations (e.g., providing regular prompts prior to transitioning tasks). Educators may benefit from learning how to modify their current universal practices (e.g., establishing routines, behavior-specific praise) to be trauma-informed. For example, evidence supports the use of strategies (e.g., positive greeting at the door) (LaBrot et al., in press; Lawson et al., in press) to connect and engage with students. If a teacher is “high fiving” to greet or praise students, it might be important for them to be aware that while most students will respond positively, a small number might be triggered by the physical contact or even flinch at a hand being raised, as these could serve as trauma cues. Thus, fostering a safe environment, especially for vulnerable youth who feel unsafe, requires educators to critically examine and challenge their own assumptions and biases regarding trauma and adversity (Hoskins et al., 2018), as well as their routine behaviors and practices with students.

Teachers may also focus on minimizing classroom distractions, modulating the intensity of stimuli (e.g., loud sounds, light brightness), and being aware of potential environmental and behavioral triggers, as children exposed to trauma may be especially sensitive to these cues. Educators may benefit from training and ongoing support on simple, evidence-informed strategies that can be creatively implemented to foster positive coping, emotion regulation, and resilience at the class-wide level. For example, they may introduce routine opportunities for the class to engage in brief relaxation (e.g., diaphragmatic breathing, progressive muscle relaxation), guided imagery, and mindfulness exercises (Duane et al., 2021; Greenberg & Harris, 2012; Khng, 2023; Obradović et al., 2021), as well as other healthy coping skills developed or adapted for young children.

As universal screening already occurs at Tier 1, administrators and educators may also consider utilizing brief screeners for stressors (e.g., familial, school) that might impact children’s functioning. Notably, the field still lacks consensus on the benefits, costs, and risks of universal trauma screening—some advocate for its use while others propose caution given the state of the science (Butcher et al., 2020; Connell et al., 2024; Eklund et al., 2018; Lang & Connell, 2017; Spence et al., 2021). Considering the need to engage in data-driven decision-making, this is a domain that warrants ongoing research.

Finally, educators can continue learning, access resources and handouts, and join collaborative and supervisory networks through the National Child Traumatic Stress Network (https://www.nctsn.org/), National Mass Violence Center (https://nmvvrc.org/), International Society for Traumatic Stress Studies (https://istss.org/), and National Association of School Psychologists (https://www.nasponline.org/resources-and-publications/resources-and-podcasts/school-safety-and-crisis/mental-health-resources/trauma, accessed on 1 August 2025), among other leading organizations in child mental health and trauma. Broadly, implementing such class-wide supports and trauma-informed adaptations may help buffer the potential effects of trauma for exposed children prior to the onset of noticeable difficulties.

### 3.2. Tier 2

In PW-PBIS, Tier 2 provides targeted relational and skill-building supports for children requiring more than universal strategies. While universal trauma screening may not yet be fully supported, targeted assessment to identify children at risk for—or already experiencing—emerging challenges associated with trauma is warranted. Various tools to assess exposure to adverse events and childhood symptoms of trauma (e.g., Denton et al., 2017; Sachser et al., 2017; Strand et al., 2005) and traumatic grief (N. Ennis et al., 2023) have been implemented in pediatric and educational settings.

Notably, after children have been identified for and begun Tier 2 supports, they should still be receiving Tier 1 supports. By requiring more individualized attention, children in Tier 2 might be assigned an increment to Tier 1 supports. For example, if all students at Tier 1 were tasked with one daily relaxation activity (2–5 min), a child in Tier 2 might be encouraged to engage in more than one relaxation activity per day and/or engage in it for a longer duration (5–10 min). Tier 2 strategies can also include class-wide group contingencies (e.g., Pasqua et al., 2021; Pokorski et al., 2017; Smith et al., 2025), teacher training in targeted classroom management skills (e.g., DeFouw et al., 2025; Gomez et al., 2021; Johnson et al., 2024; LaBrot et al., 2024), and small group interventions (e.g., Daunic et al., 2013; Stanton-Chapman et al., 2014). While effective in traditional PW-PBIS, these approaches can be adapted for trauma-exposed children by emphasizing social–emotional learning and emotion regulation.

Further, relational support (e.g., daily check-in/check-out with a trusted adult; LaBrot et al., 2016; LeBel et al., 2013) may be particularly critical, as children who have experienced trauma often require more explicit and sustained attention to build trust (Hackney et al., 2024). Additional strategies may include incorporating specific “safe spaces” for children who need a moment to self-regulate, regularly asking questions and offering choices, and reminding children of potential coping strategies they can use (e.g., breathing, counting, drawing). Creating a trauma-informed educational environment also involves systematically educating staff, students, caregivers, and stakeholders about trauma-related policies and procedures, as well as establishing clear pathways for accessing resources.

### 3.3. Tier 3

Tier 3 emphasizes individualized, intensive supports for children who have been identified as having social–emotional or behavioral difficulties. When considering a trauma-informed approach, in addition to the strategies highlighted in Tiers 1 and 2, this would include the integration of trauma-specific approaches into behavior plans, as well as collaboration with mental health professionals to best address the child’s trauma-related difficulties. Similarly, adaptations to individualized education and safety plans will also be necessary to ensure plans account for trauma histories and are appropriate given the child’s experiences. Some children may need individual behavior plans that lean away from restrictive environments (Hunter et al., 2021). Similarly, while setting boundaries and upholding rules are important, we caution against engaging in punitive measures to respond to children’s trauma symptoms, which could be re-traumatizing. In a trauma-informed approach, Tier 3 may include connecting children with school personnel able to provide additional supports, such as behavior specialists, counselors, or other health or mental health supports. If access to the level of care needed is not available within the school setting, referrals to external providers (e.g., clinical psychologists) with expertise in treating early childhood trauma are warranted. Should a child present with significant trauma-related symptoms, the child may benefit from a trauma-specific therapeutic modality. While several have been proposed, one trauma-specific intervention for children has the strongest evidence to support its use.

Trauma-focused cognitive behavioral therapy (TF-CBT; Cohen et al., 2018) is an evidence-based treatment that addresses posttraumatic stress symptomology experienced by youth aged 3–18 years old. TF-CBT takes a components-based approach to the treatment of trauma in children and their nonoffending caregivers. The treatment comprises three phases: stabilization, trauma narration and processing, and integration and consolidation of skills. The treatment is typically completed in 12–20 sessions. As mentioned, many children are exposed to events that may impact their well-being, potentially resulting in socioemotional and behavioral concerns. TF-CBT does not require children to meet diagnostic criteria for PTSD to receive treatment (National TF-CBT Certification Program, 2025), though the child must have experienced or witnessed a PTE for treatment to be warranted. Research supports TF-CBT as a well-established evidence-based intervention for trauma-exposed youth, recommending it as a first-line treatment (de Arellano et al., 2014; Thielemann et al., 2022).

## 4. Trauma-Informed PW-PBIS in Early Childhood Education: Illustrating Our Approach

A local university institutional review board approved using data from this case for scholarly purposes. The child’s parent provided informed consent, and the child provided assent.

### 4.1. Context of the Case Example

This case took place at a Head Start agency located in the southeastern United States. Agency child demographics included 85% Black, 7% White, 5% Latine, 4% multiracial, and 3% English language learners. Each site utilized PW-PBIS as described above to address the behavioral and mental health needs of the children. Additionally, teachers completed Behavior Incident Reports (BIRs) as needed, and the behavior consultants tracked these data to provide needed services (e.g., teacher consultation, functional behavior assessments). Two doctoral-level school psychology graduate students served as behavior consultants for this site under the supervision of a licensed psychologist: a 36-year-old White female and a 25-year-old Latina female. Both consultants managed the PW-PBIS multitiered systems of support, conducted social–emotional and developmental screeners, provided system-wide and teacher consultation, conducted functional behavior assessments, created behavior plans, provided parent consultation and direct services, and provided program-wide professional development. This case example was selected as it was representative of our pilot efforts to integrate a trauma-informed approach at each tier of the PW-PBIS model in an early childhood education context.

### 4.2. Case Introduction

Tyrese (pseudonym) was a 4-year-old Black male child who was referred for behavioral services due to elevated levels of peer conflict (i.e., social isolation, name-calling) and not following teachers’ instructions. The teacher and assistant teacher struggled to respond to these instances of challenging behavior. Tyrese’s teachers described utilizing precorrections, behavior-specific praise, planned ignoring, and the use of contingent rewards with little to no change in Tyrese’s behavior.

Upon further evaluation, it was identified that Tyrese had been exposed to a traumatic event. Specifically, Tyrese witnessed domestic violence between his mother and father and witnessed the death of his father after he was shot. Tyrese’s mother and teacher reported that Tyrese frequently asked questions about his father’s death and engaged in aggressive play with his peers. Tyrese’s mother described that Tyrese experienced sudden episodes of dysregulation (e.g., crying, tantrums, irritability) and demonstrated an increase in emotional symptoms around male children and adults. A behavior incident report was written by Tyrese’s teacher stating that Tyrese brought a butter knife to school and expressed intention to hurt one of his peers. A school-based threat assessment was conducted, and risk was considered low. After this incident, the Child and Adolescent Trauma Screen ([CATS]; Sachser et al., 2017) was conducted, revealing probable PTSD (see Table 1). The CATS is a free, brief, and accessible trauma screener for ages three to 17 that aligns with the Diagnostic and Statistical Manual of Mental Disorders, Fifth Edition (DSM-5) criteria for PTSD (Sachser et al., 2017). The CATS was completed by Tyrese’s mother and was administered via interview by the consultant. Specific items endorsed included acting, playing, or feeling as if the traumatic event is happening right now; feeling emotionally upset when reminded of the traumatic event; trying not to remember, talk, or think about the traumatic event; an increase in emotional states; acting socially withdrawn; a reduction in expressing positive feelings; and being overly alert or on guard. Anecdotally, prior to intervention, Tyrese’s mother described that he was withdrawn and sad, often asked questions regarding his father’s death, and reported concerns regarding his mother’s health. His teacher described him as “jumpy” and “irritable.” Per their reports, Tyrese appeared withdrawn from his peers and refused to talk to others when dysregulated, almost every day.

### 4.3. Case Conceptualization

Tyrese’s symptoms were consistent with a diagnosis of PTSD. Additionally, Tyrese’s teachers described limited response to typical Tier 1 supports. Given that Tyrese’s symptoms were present across the school and home environment, it became clear that a trauma-informed approach was warranted to address his concerns. As such, behavioral consultants conceptualized exposure to trauma as a primary function of Tyrese’s challenging behaviors. A functional behavioral assessment revealed the hypothesized function of his behavior as access to attention (i.e., to seek help, support, and comfort from his teachers).

### 4.4. Course of Treatment and Assessment of Progress

#### 4.4.1. Tier 1

Tyrese’s teachers reported that they struggled to address negative peer interactions with not only Tyrese but also with several other children in the classroom. Therefore, the behavior consultants trained the teachers to provide 5 min, class-wide social skills lessons three times a week. The skills taught included sharing and turn-taking, using kind words, and working together. In tandem, a class-wide group contingency was implemented in which the teacher provided behavior-specific praise and rewards if the entire class was observed to engage in positive peer interactions. After four weeks of implementation, there was a 30% increase in class-wide positive interactions as measured by direct behavior observations. This class-wide intervention was considered trauma-informed, as the focus was on skill-building, relationship-building, and positive reinforcement (Joseph et al., 2025; Rajaraman et al., 2022). This supported the building of safety and trust within Tyrese’s experiences in the school environment and opportunities to contact natural reinforcement for effective use of prosocial skills. Following class-wide intervention, the teachers reported that Tyrese no longer engaged in negative interactions with his peers. However, BIRS data indicated Tyrese continued to struggle with initiating these interactions, frequently played alone, and engaged in power struggles with his teacher due to difficulties following instructions and answering questions. Tyrese’s mother also disclosed the potentially traumatic event. These data indicated a need for Tier 2 supports.

#### 4.4.2. Tier 2

Tier 2 supports were delivered about three weeks after Tier 1 supports began. Given previous power struggles and Tyrese’s hesitancy to trust adults, it was clear that establishing a safe and supportive relationship between Tyrese and his teacher was necessary. In anticipation of potential escalation to Tier 3, a brief functional assessment was conducted and revealed that Tyrese engaged in these behaviors to seek attention from preferred adults (i.e., help, comfort, reassurance). So, the behavior consultant provided teacher training on the use of positive interaction skills as outlined in Teacher-Child Interaction Training ([TCIT]; Lyon et al., 2009). Positive interaction skills were delivered by his teacher for at least five minutes each day during center activities. Specifically, the teacher provided a choice of activities and/or joined an ongoing play activity and enthusiastically provided praise for positive behaviors, reflected Tyrese’s language, and imitated/described Tyrese’s play actions.

In addition to the daily implementation of time-in, Tyrese’s assistant teacher implemented early childhood modified Check-in/Check-out ([CICO]; LaBrot et al., 2016) each morning/afternoon. CICO was modified to be trauma-informed by incorporating explicit social–emotional teaching to increase Tyrese’s ability to effectively communicate and cope with his emotions, as opposed to a focus on classroom behaviors. The behavior consultant provided a box (i.e., a pencil case containing visuals of emotions and coping strategies for Tyrese to choose from) in which Tyrese could choose the relevant emotion (using cartoon faces) and potential coping strategies (e.g., drinking water, taking deep breaths, squeezing a soft toy, talking to his teacher) given the emotion. Each morning, Tyrese’s assistant teacher provided a greeting and encouragement (e.g., “Good morning, Tyrese! It’s going to be a great day!”), directed Tyrese to choose an emotion and an appropriate coping strategy, if needed, from the box as described above. Then, the teacher reminded Tyrese of his goals/potential for reinforcement. At the end of the day, Tyrese’s teacher provided feedback regarding his goals using a daily behavior report card (DBRC) and communicated progress with his caregiver. The DBRC served as an intervention and progress monitoring tool to assess response to intervention. Throughout this process, parent training was provided regarding the use of positive interaction skills at home, effective instructions, and positive reinforcement strategies given for his behavior each day, as reported by Tyrese’s teacher on the DBRC. Due to persistent emotional and behavioral symptoms, the CATS was administered, which revealed potential PTSD symptoms, which then triggered the implementation of Tier 3 supports.

#### 4.4.3. Tier 3

The behavior consultant increased the intensity of intervention by providing in-classroom support during free play and pull-out sessions about twice a week for 15 min at a time to deliver Trauma-Focused Cognitive Behavioral Therapy (TF-CBT). The first four pull-out sessions were focused on rapport-building, as Tyrese had historically struggled to interact with new adults. Adaptations to TF-CBT were incorporated for developmental appropriateness, such as the use of visuals, read-alouds, and card games to facilitate psychoeducation. For example, the consultant utilized picture books to support Tyrese’s ability to accurately label basic emotions and identify possible thoughts and actions relevant to the character. Card games were adapted to increase engagement, incorporating a play-based approach, by adjusting the rules to relevant scenarios and potential thoughts/feelings/actions. Visuals were used to increase understanding (e.g., emotion choice board, stress thermometer, pictures depicting thoughts, feelings, and actions).

Tyrese was instructed to identify emotions, thoughts, and behaviors (i.e., actions) in isolation, given a scenario or story, rather than connecting all three simultaneously (i.e., “Given this scenario, what might he be thinking?” or “What could he have been feeling?”). The in vivo mastery phase of TF-CBT was abbreviated due to barriers in the school setting and inappropriateness given the traumatic event. However, the consultant noticed that Tyrese avoided talking about feeling sad and immediately and frequently changed the topic of conversation in session when asked about feeling sad. For this reason, Tyrese’s teacher was directed to facilitate discussion of difficult feelings due to a history of avoidance. The trauma narrative was completed in five sessions, including two conjoint sessions with Tyrese’s mother. Tyrese chose to draw pictures of each chapter, and the behavior consultant transcribed his description of the event. The chapters were titled as follows: (1) all about me, (2) my safe spaces, (3) my family, (4) my feelings, (5) my dad, (6) what happened next, (7) how I feel now, and (8) how I can stay safe. Tyrese read the story to his mother during the conjoint sessions and practiced coping strategies together. Tyrese’s mother maintained a supportive expression during these sessions but expressed individually with the consultant the difficulty of hearing Tyrese’s story, given her experiences with domestic violence.

Regarding parent collaboration in TF-CBT, the behavior consultant utilized telephone, telehealth, and asynchronous sessions with Tyrese’s mother to support psychoeducation, parent training, and effective practice/implementation of strategies at home due to Tyrese’s mother’s work schedule. However, conjoint sessions were held in person after school hours. Further, parent training was provided regarding Tyrese’s avoidance of difficult emotions. This involved psychoeducation regarding functions of behavior and adjustment of Tyrese’s mother’s response to crying (i.e., how to teach emotion identification, functional communication training, and de-escalation strategies).

Assessment of response to intervention was conducted throughout each tier of Tyrese’s treatment. An additional CATS screener was conducted following TF-CBT. This CATS screener determined Normal, Not Clinically Elevated PTSD Symptoms, with a score of 11. Also, Tyrese’s mother and teacher reported that Tyrese was no longer withdrawn and more frequently engaged with his peers. Additionally, the first three DBRCs completed in Tier 2 revealed that Tyrese was sad and withdrawn most of the time, and the final three DBRCs completed in Tier 3 revealed that Tyrese was calm and/or active, reflecting a decrease in withdrawal. However, the teacher reported continued irritability. Subjective Units of Distress (SUDS) were recorded at the beginning, middle, and end of each session to measure emotional intensity and effectiveness of exposures (i.e., trauma narrative; Benjamin et al., 2010; Kiyimba & O’Reilly, 2020). A decrease in SUDS during sessions was observed (4.5 to 3 on a scale of 1 to 5). Anecdotally, following intervention, Tyrese’s mother expressed better understanding of his symptoms and their origin and noted an increase in energy, greater mood and behavior regulation, and described that Tyrese “verbally expressed his emotions instead of getting quiet” and “sometimes even prompted me to take deep breaths.” Tyrese’s teacher reported that Tyrese engaged with his peers more often and expressed his wants and needs rather than “shutting down.” Overall, both in reduction in symptoms and increased functionality, Tyrese responded well to the intervention, above and beyond what would have been expected with standard implementation of PW-PBIS.

## 5. Discussion

Because early childhood adversity is highly prevalent and can confer substantial risks to young children (Liming & Grube, 2018), early childhood education programs may be uniquely poised to help address this public health concern by supporting early identification and prevention. However, many school professionals report a lack of training, resources, and support to adequately meet the needs of children exposed to trauma. In this paper, we described the plight of preschool children exposed to adversity, presented trauma-informed strategies at each tier within PW-PBIS, and highlighted case examples from a pilot implementation of this model.

The trauma-informed practices described here were guided by empirical literature and the authors’ research, clinical, and supervision experiences with children in early education contexts and children impacted by trauma. Among strategies highlighted included ongoing training, support, and resources for early educators on: adverse events; identifying signs of neglect and abuse; identifying common symptoms, triggers, and reactions; evidence-informed coping and regulation strategies; integrating trauma-informed strategies into existing practice; establishing safe spaces; providing clear instructions and expectations; modeling and fostering empathy; promoting prosocial behaviors; minimizing distractions and stimuli; screening (when appropriate) to determine who may benefit from more intensive supports; developing trauma-informed policies and procedures; behavioral and mental health consultation; triage and referral processes; implementing trauma-specific psychosocial interventions (e.g., TF-CBT); and seeking additional learning opportunities and resources (e.g., NCTSN; ISTSS; NASP).

This paper presented illustrative examples of the model from our work in integrating trauma-informed strategies in an early childhood education setting. We described specific steps at each PW-PBIS level, including class-wide strategies (Tier 1) and individualized supports at Tiers 2 and 3. Services for Tyrese (child’s pseudonym) were provided by a behavior consultant and a school psychology trainee (Author 3) embedded in a local Head Start, supervised by a licensed psychologist (Author 2). Initially guided by standard practices, the consultant recognized Tyrese required additional support, one not typically implemented in traditional PW-PBIS. Tyrese had been exposed to violence and traumatic loss at home. As such, the consultant sought ongoing consultation from a licensed psychologist (Author 1) trained in trauma-informed care.

Given Tyrese’s concerns, the consultant engaged in trauma-informed modifications at Tiers 1 and 2 and more intensive Tier 3 intervention (e.g., TF-CBT). We chose TF-CBT—it has high empirical support, it is modular and flexible, and it incorporates individual and conjoint sessions (Thielemann et al., 2022). TF-CBT seemed especially promising with its modifications for younger children and utility for traumatic loss (Cohen et al., 2018). We collected progress data—observing significant decreases in Tyrese’s trauma-related challenges at school and home and increases in well-being and functioning. Overall, we considered Tyrese’s case, as well as our pilot integration of trauma-informed practices in this Head Start, a success. While promising, more research is needed prior to implementing this trauma-informed model on a wider scale in early childhood PW-PBIS contexts. We discuss implications for practice and research, including implementation considerations and recommendations.

### Considerations, Limitations, and Implications

By attuning to how exposure to adverse conditions influences the functioning of children they serve, educators may become better equipped at identifying signs of trauma, reducing the risk of re-traumatization, and mitigating long-term negative consequences. To equitably address student needs, it is important to consider factors beyond the individual, such as cultural, classroom, program-wide, and societal expectations. Without doing so, educators and providers may hypothesize a function of behavior and apply the related strategies (e.g., overuse of extinction, planned ignoring, unintended exposures) that may inadvertently worsen potential trauma-related adjustment problems. Importantly, we also encourage educators to work on minimizing unintentional exposures by avoiding the placement of a child in a potentially triggering situation or environment without the tools to properly cope (Cummings et al., 2017).

In identifying children who may benefit from targeted attention, even with universal screening, it is possible to miss when a child has been exposed to a PTE or is experiencing posttraumatic challenges. Several reasons might account for inconsistent reporting or underreporting (Skar et al., 2021). Familial attitudes and cultural factors could play a role in inhibiting disclosures (Perrigo et al., 2024). It is important to take a trauma-assumed approach and involve possible stakeholders in the problem-solving process (e.g., collaborate with caregivers and other support professionals to creatively identify solutions). When a child has experienced trauma, it is likely that caregivers have as well (Wilcoxon et al., 2021). In our case example, Tyrese’s mother experienced domestic violence and struggled with chronic health issues, which can be traumatic for children (Cooley & Taussig, 2022). For Tyrese to continue to grow and learn the skills needed to cope with everyday events, community collaboration was key. Providing caregivers with outside referrals for therapy services is crucial to increasing the capacity for change, in addition to conjoint child-caregiver sessions when possible. Providers should consider flexible forms of communication with caregivers to enhance access to resources and service utilization; for example, parent sessions could be completed before or after school or via telehealth using HIPAA-compliant platforms (Villalobos et al., 2023).

To ensure greater impact when implementing PW-PBIS, special considerations must be taken regarding relevant policies and procedures. Several key players contribute to establishing and managing trauma-informed systems, including educators and school staff, families and caregivers, and agency leadership. To successfully embed trauma-informed practices and maintain long-term sustainability, it is essential that all involved parties are equipped with proper training and resources (Wassink-de Stigter et al., 2022). Among these, early childhood educators are in an ideal position to support children exposed to trauma and mitigate potentially harmful developmental outcomes (Loomis, 2018).

Historically, teachers report a lack of confidence in their knowledge on how to provide an appropriate response and support for a child exposed to trauma, often unaware of protocols within their schools (Alisic, 2012). To bolster teacher confidence when approaching sensitive topics (e.g., trauma, grief, crises), schools may consider adopting clear guidelines for what the teacher’s role should include. It may help to convey the threshold for where teacher responsibility ends and specialized professional assistance (e.g., school psychologist) begins (Alisic, 2012). Further, teachers report needing specific training regarding trauma-related terminology, signs and symptoms of trauma, and strategies for self-care (Sun et al., 2024a). Training may cover responding to trauma-related behaviors, establishing safe learning environments, teaching coping techniques for students, and understanding indicators of recovery or need for more support (Alisic, 2012). Educators may also learn how post-trauma reactions could be misattributed to other problems, like inattentiveness, oppositional defiance, or learning disabilities. Further, teachers may benefit from receiving training on clear role guidelines and expectations, protocols for having difficult conversations with children and families, and implementation of school-wide social–emotional learning curriculum (Brunzell et al., 2015; Sun et al., 2024b).

Young children with significant trauma history and elevated posttraumatic symptoms may require connecting families to external resources or community mental health services. Partnerships with families begin with developing strong rapport and trusting relationships to foster a safe environment (Berger et al., 2022). Teachers and providers can better develop plans to address children’s challenges when caregivers feel comfortable disclosing information about their child’s environment and experiences. Because many are enrolled in school—estimates suggest 59% of 3- to 5-year-olds attend school (National Center for Education Statistics)—teachers are typically one of the few adults outside the home interfacing with these children. This means teachers are likely the first line of defense in recognizing symptoms and serving as a liaison between families and professional resources when warranted. This emphasizes the need for teachers to be aware of and connected with mental and behavioral health providers, especially those operating through a trauma-informed lens. School personnel benefit greatly from learning about external trauma resources and services and developing a clear referral system for children and families when needed. The use of external resources recommended to families by early childhood professionals heavily relies on caregivers; thus, calls have been made to increase understanding of how to best incorporate families in the referral process (Brown et al., 2022).

Another important consideration is developing a better understanding of the link between trauma and systemic adversity (Brown et al., 2022). Calls have been made for future in-service and pre-service training for educators to include a greater focus on performance feedback with a trauma-informed lens. Educators may strive to provide responsive trauma-informed care—trauma is more likely to be experienced by students of color, minoritized backgrounds, and those living in poverty (Sacks & Murphey, 2018). It may be helpful to identify students at greater risk for trauma-related exposure, support distinctive efforts to address the trauma, and adjust approaches and referrals as needed (Brown et al., 2022). Notably, some authors have cautioned against the use of PBIS, suggesting its tenets are inconsistent with culturally responsive or trauma-informed approaches (Kim & Venet, 2025). While some criticisms may be valid, we consider the potential costs and risks of overhauling systems already in place, rather than the pragmatics of leveraging and modifying existing resources and frameworks to better suit the needs of young children already embedded in those systems.

Trauma-informed interventions have been implemented at the universal (Tier 1) and selective levels (Tier 2) in some school-based settings. In the aftermath of crises (e.g., Hurricane Katrina), Psychological First Aid (PFA) has been used to enhance children’s sense of safety, determine basic needs, connect families to supportive resources, and promote hope (Ruzek et al., 2007; L. Wang et al., 2021). Skills for Psychological Recovery (SPR) is considered a targeted intervention, aiming to enhance resilience in children and families to cope with the effects of trauma and disasters (Wade et al., 2014). As a more intensive support (Tier 2), SPR emphasizes problem-solving, emotion regulation, coping mechanisms, decision-making, positive thinking, social support, and self-care. PFA and SPR were developed for sustainability, focusing on skills and training for persons (e.g., first responders) not formally trained in mental health. Preliminary work suggests teachers can be trained in these models and implement learned strategies (e.g., coping skills) at the class and individual levels (Orengo-Aguayo et al., 2019). However, more research is warranted on implementation outcomes of these in preschool settings.

## 6. Conclusions

In this paper, we discussed the needs of young children exposed to adversity and trauma, highlighting common reactions and risk factors. We advocated for incorporating a trauma-informed lens into early childhood education, specifically within PW-PBIS. At each tier, we described trauma-informed strategies and promising intervention programs that may be considered for implementation. We presented a pilot case on our integrated trauma-sensitive model at a local Head Start. Results were promising—the child showed significant improvements in overall well-being, and we found preliminary support from site staff on the model’s acceptability.

In sum, early childhood educators are in a position to support early identification and support efforts for children who have undergone traumatic experiences, especially within a well-run trauma-informed system (Berger et al., 2022). Due to the scarcity of trauma-informed education within standard teacher training, organizations should prioritize incorporating relevant supports to meet the needs of students and educators. The field is urgently lacking research on trauma-informed care in early childhood contexts. To mitigate risk in vulnerable youth, we make a call to researchers, practitioners, and administrators to expand research on trauma-informed practices in early childhood programs. In closing, this pilot case using a trauma-sensitive lens within an existing PW-PBIS framework supports the promise of integrating trauma-informed care into early childhood education contexts.

## Figures and Tables

**Table 1:** Pre-post CATS screener.

Time of Year	Score	Cut-Score	Description
Fall 2024	20	16	Probable PTSD
Spring 2025	11	15	Normal, not clinically elevated

## Data Availability

The datasets presented in this article are not readily available because they are derived from real clinical client data and were only granted permission to share in this manuscript as part of a case study.

## References

[B1-behavsci-15-01640] Alisic E. (2012). Teachers’ perspectives on providing support to children after trauma: A qualitative study. School Psychology Quarterly.

[B2-behavsci-15-01640] Alisic E., Bus M., Dulack W., Pennings L., Splinter J. (2012). Teachers’ experiences supporting children after traumatic exposure. Journal of Traumatic Stress.

[B3-behavsci-15-01640] Alisic E., Van der Schoot T. A., van Ginkel J. R., Kleber R. J. (2008). Looking beyond posttraumatic stress disorder in children: Posttraumatic stress reactions, posttraumatic growth, and quality of life in a general population sample. Journal of Clinical Psychiatry.

[B4-behavsci-15-01640] Andersen S. B., Karstoft K. I., Bertelsen M., Madsen T. (2014). Original research latent trajectories of trauma symptoms and resilience: The 3-year longitudinal prospective USPER study. Journal of Clinical Psychiatry.

[B5-behavsci-15-01640] Bartlett J. D. (2021). Trauma-informed practices in early childhood education.

[B6-behavsci-15-01640] Bartlett J. D., Smith S. (2019). The role of early care and education in addressing early childhood trauma. American Journal of Community Psychology.

[B7-behavsci-15-01640] Benjamin C. L., O’Neil K. A., Crawley S. A., Beidas R. S., Coles M., Kendall P. C. (2010). Patterns and predictors of subjective units of distress in anxious youth. Behavioural and Cognitive Psychotherapy.

[B8-behavsci-15-01640] Benjet C., Bromet E., Karam E. G., Kessler R. C., McLaughlin K. A., Ruscio A. M., Shahly V., Stein D. J., Petukhova M., Hill E., Alonso J., Atwoli L., Bunting B., Bruffaerts R., Caldas-de-Almeida J. M., de Girolamo G., Florescu S., Gureje O., Huang Y., Koenen K. C. (2016). The epidemiology of traumatic event exposure worldwide: Results from the World Mental Health Survey Consortium. Psychological Medicine.

[B9-behavsci-15-01640] Berger E. (2019). Multi-tiered approaches to trauma-informed care in schools: A systematic review. School Mental Health.

[B10-behavsci-15-01640] Berger E., O’Donohue K., La C., Quinones G., Barnes M. (2022). Early childhood professionals’ perspectives on dealing with trauma of children. School Mental Health.

[B11-behavsci-15-01640] Briggs-Gowan M. J., Ford J. D., Fraleigh L., McCarthy K., Carter A. S. (2010). Prevalence of exposure to potentially traumatic events in a healthy birth cohort of very young children in the northeastern United States. Journal of Traumatic Stress.

[B12-behavsci-15-01640] Brown E. C., Freedle A., Hurless N. L., Miller R. D., Martin C., Paul Z. A. (2022). Preparing teacher candidates for trauma-informed practices. Urban Education.

[B13-behavsci-15-01640] Brunzell T., Waters L., Stokes H. (2015). Teaching with strengths in trauma-affected students: A new approach to healing and growth in the classroom. American Journal of Orthopsychiatry.

[B14-behavsci-15-01640] Butcher R. L., Jankowski M. K., Slade E. D. (2020). The costs of implementing and sustaining a trauma and mental health screening tool in a state child welfare system. Children and Youth Services Review.

[B15-behavsci-15-01640] Cain K. S., Meyer S. C., Cummer E., Patel K. K., Casacchia N. J., Montez K., Palakshappa D., Brown C. L. (2022). Association of food insecurity with mental health outcomes in parents and children. Academic Pediatrics.

[B16-behavsci-15-01640] Center for Health Care Strategies (2022). What is trauma-informed care? *Trauma-Informed Care Implementation Resource Center*.

[B17-behavsci-15-01640] Choi J.-K., Wang D., Jackson A. P. (2019). Adverse experiences in early childhood and their longitudinal impact on later behavioral problems of children living in poverty. Child Abuse & Neglect.

[B18-behavsci-15-01640] Chudzik M., Corr C., Fisher K. W. (2024). Trauma-informed care: The professional development needs of early childhood special education teachers. Journal of Early Intervention.

[B19-behavsci-15-01640] Clarkson Freeman P. A. (2014). Prevalence and relationship between adverse childhood experiences and child behavior among young children. Infant Mental Health Journal: Infancy and Early Childhood.

[B20-behavsci-15-01640] Cohen J. A., Deblinger E., Mannarino A. P. (2018). Trauma-focused cognitive behavioral therapy for children and families. Psychotherapy Research: Journal of the Society for Psychotherapy Research.

[B21-behavsci-15-01640] Connell C. M., Swanson A. S., Genovese M., Lang J. M. (2024). Effects of child trauma screening on trauma-informed multidisciplinary evaluation and service planning in the child welfare system. Journal of Traumatic Stress.

[B22-behavsci-15-01640] Cook A., Spinazzola J., Ford J., Lanktree C., Blaustein M., Cloitre M., van der Kolk B. (2005). Complex trauma. Psychiatric Annals.

[B23-behavsci-15-01640] Cooley J. L., Taussig H. N. (2022). Anger and attention problems as mechanisms linking maltreatment subtypes and witnessed violence to social functioning among children in out-of-home care. Child Maltreatment.

[B24-behavsci-15-01640] Copeland W. E., Keeler G., Angold A., Costello E. J. (2007). Traumatic events and posttraumatic stress in childhood. Archives of General Psychiatry.

[B25-behavsci-15-01640] Cprek S. E., Williamson L. H., McDaniel H., Brase R., Williams C. M. (2020). Adverse Childhood Experiences (ACEs) and risk of childhood delays in children ages 1–5. Child and Adolescent Social Work Journal.

[B26-behavsci-15-01640] Cummings K. P., Addante S., Swindell J., Meadan H. (2017). Creating supportive environments for children who have had exposure to traumatic events. Journal of Child and Family Studies.

[B27-behavsci-15-01640] Daníelsdóttir H. B., Aspelund T., Shen Q., Halldorsdottir T., Jakobsdóttir J., Song H., Lu D., Kuja-Halkola R., Larsson H., Fall K., Magnusson P. K. E., Fang F., Bergstedt J., Valdimarsdóttir U. A. (2024). Adverse childhood experiences and adult mental health outcomes. JAMA Psychiatry.

[B28-behavsci-15-01640] Daunic A., Corbett N., Smith S., Barnes T., Santiago-Poventud L., Chalfant P., Pitts D., Gleaton J. (2013). Brief report: Integrating social-emotional learning with literacy instruction: An intervention for children at risk for emotional and behavioral disorders. Behavioral Disorders.

[B29-behavsci-15-01640] de Arellano M. A. R., Lyman D. R., Jobe-Shields L., George P., Dougherty R. H., Daniels A. S., Ghose S. S., Huang L., Delphin-Rittmon M. E. (2014). Trauma-focused cognitive-behavioral therapy for children and adolescents: Assessing the evidence. Psychiatric Services.

[B30-behavsci-15-01640] DeFouw E. R., LaBrot Z. C., Garza B. D., Johnson C., Cato T., McVay K., Sweaks A. (2025). Short clips, big impacts: Enhancing preschool teachers’ effective instruction delivery with video modeling reels. Journal of Behavioral Education.

[B31-behavsci-15-01640] Denton R., Frogley C., Jackson S., John M., Querstret D. (2017). The assessment of developmental trauma in children and adolescents: A systematic review. Clinical Child Psychology and Psychiatry.

[B32-behavsci-15-01640] Downey C., Crummy A. (2022). The impact of childhood trauma on children’s wellbeing and adult behavior. European Journal of Trauma & Dissociation.

[B33-behavsci-15-01640] Duane A., Casimir A. E., Mims L. C., Kaler-Jones C., Simmons D. (2021). Beyond deep breathing: A new vision for equitable, culturally responsive, and trauma-informed mindfulness practice. Middle School Journal.

[B34-behavsci-15-01640] Dye H. (2018). The impact and long-term effects of childhood trauma. Journal of Human Behavior in the Social Environment.

[B35-behavsci-15-01640] Eber L., Barrett S., Scheel N., Flammini A., Pohlman K. (2020). Integrating a trauma-informed approach within a PBIS framework.

[B36-behavsci-15-01640] Eklund K., Rossen E., Koriakin T., Chafouleas S. M., Resnick C. (2018). A systematic review of trauma screening measures for children and adolescents. School Psychology Quarterly.

[B37-behavsci-15-01640] Ennis N., Pastrana F. A., Moreland A. D., Davies F., DelMas S., Rheingold A. (2023). Assessment tools for children who experience traumatic loss: A systematic review. Trauma, Violence, & Abuse.

[B38-behavsci-15-01640] Ennis R. P., Royer D. J., Lane K. L., Griffith C. E. (2017). A systematic review of precorrection in PK-12 settings. Education and Treatment of Children.

[B39-behavsci-15-01640] Farmer T. W., McAuliffe Lines M., Hamm J. V. (2011). Revealing the invisible hand: The role of teachers in children’s peer experiences. Journal of Applied Developmental Psychology.

[B40-behavsci-15-01640] Ferrara A. M., Panlilio C. C., Tirrell-Corbin C. (2023). Exploring school professionals’ definitions of childhood trauma. Journal of Child & Adolescent Trauma.

[B41-behavsci-15-01640] Fox L., Carta J., Strain P. S., Dunlap G., Hemmeter M. L. (2010). Response to intervention and the pyramid model. Infants & Young Children.

[B42-behavsci-15-01640] Galatzer-Levy I. R., Huang S. H., Bonanno G. A. (2018). Trajectories of resilience and dysfunction following potential trauma: A review and statistical evaluation. Clinical Psychology Review.

[B43-behavsci-15-01640] Gautam N., Rahman M. M., Khanam R. (2024). Adverse childhood experiences and externalizing, internalizing, and prosocial behaviors in children and adolescents: A longitudinal study. Journal of Affective Disorders.

[B44-behavsci-15-01640] Gomez L., Barton E. E., Winchester C., Locchetta B. (2021). Effects of email performance feedback on teachers’ use of play expansions. Journal of Early Intervention.

[B45-behavsci-15-01640] Greenberg M. T., Harris A. R. (2012). Nurturing mindfulness in children and youth: Current state of research. Child Development Perspectives.

[B46-behavsci-15-01640] Hackney A. J., Jolivette K., Sanders S. (2024). Integrating trauma-informed practices into check-in/check-out for use in alternative education settings. Intervention in School and Clinic.

[B47-behavsci-15-01640] Haczkewicz K. M., Shahid S., Finnegan H. A., Monnin C., Cameron C. D., Gallant N. L. (2024). Adverse childhood experiences (ACEs), resilience, and outcomes in older adulthood: A scoping review. Child Abuse & Neglect.

[B48-behavsci-15-01640] Hart H., Rubia K. (2012). Neuroimaging of child abuse: A critical review. Frontiers in Human Neuroscience.

[B49-behavsci-15-01640] Hemmeter M. L., Fox L., Jack S., Broyles L. (2007). A program-wide model of positive behavior support in early childhood settings. Journal of Early Interventions.

[B50-behavsci-15-01640] Hoover S. A., Sapere H., Lang J. M., Nadeem E., Dean K. L., Vona P. (2018). Statewide implementation of an evidence-based trauma intervention in schools. School Psychology Quarterly.

[B51-behavsci-15-01640] Hoskins D., Duncan L. G., Moskowitz J. T., Ordóñez A. E. (2018). Positive Adaptations for Trauma and Healing (PATH), a pilot study of group therapy with Latino youth. Psychological Trauma: Theory, Research, Practice, and Policy.

[B52-behavsci-15-01640] Hunter W., Taylor J. C., Bester M., Nichols S., Panlilio C. (2021). Considerations for incorporating trauma-informed care content within special education teacher preparation and professional development programs. Journal of Special Education Preparation.

[B53-behavsci-15-01640] Jimenez M. E., Wade R., Lin Y., Morrow L. M., Reichman N. E. (2016). Adverse experiences in early childhood and kindergarten outcomes. Pediatrics.

[B54-behavsci-15-01640] Johnson C. N., Cato T. A., LaBrot Z. C., DeFouw E. R. (2024). Evaluation of emailed prompts to promote generalization and maintenance of preschool teachers’ effective instruction delivery. Behavioral Interventions.

[B55-behavsci-15-01640] Joseph J., Watson D., Sayles J. (2025). Leading with compassion: Building trauma-informed Pyramid Model programs.

[B56-behavsci-15-01640] Khng K. H. (2023). Deep breathing and mindfulness: Simple techniques to promote students’ self-regulation and well-being from the inside out. Positive psychology and positive education in Asia: Understanding and fostering well-being in schools.

[B57-behavsci-15-01640] Kim R. M., Venet A. S. (2025). Unsnarling PBIS and trauma-informed education. Urban Education.

[B58-behavsci-15-01640] Kiyimba N., O’Reilly M. (2020). The clinical use of Subjective Units of Distress scales (SUDs) in child mental health assessments: A thematic evaluation. Journal of Mental Health.

[B59-behavsci-15-01640] LaBrot Z. C., Dufrene B. A., Pasqua J., Radley K. C., Olmi D. J., Bates-Brantley K., Murphy A. (2018). A comparison of two function-based interventions: NCR vs. DRO in preschool classrooms. Preventing School Failure: Alternative Education for Children and Youth.

[B60-behavsci-15-01640] LaBrot Z. C., Dufrene B. A., Radley K., Pasqua J. (2016). Evaluation of a modified check-in/check-out intervention for young children. Perspectives on Early Childhood Psychology and Education.

[B61-behavsci-15-01640] LaBrot Z. C., Garza B. D., Rodriguez L., Abril Ochoa A., DeFouw E. R. (in press). Impact of behavioral skills training on assistant teachers’ use of positive greetings at the door. Journal of Behavioral Education.

[B62-behavsci-15-01640] LaBrot Z. C., Johnson C. N., Maxime E., Cobek C., Dufrene B. A. (2024). In situ training to improve early childhood teachers’ effective instruction delivery. Journal of Educational and Psychological Consultation.

[B63-behavsci-15-01640] Lai B. S., Lewis R., Livings M. S., La Greca A. M., Esnard A. (2017). Posttraumatic stress symptom trajectories among children after disaster exposure: A review. Journal of Traumatic Stress.

[B64-behavsci-15-01640] Lang J. M., Connell C. M. (2017). Development and validation of a brief trauma screening measure for children: The child trauma screen. Psychological Trauma: Theory, Research, Practice, and Policy.

[B65-behavsci-15-01640] Lawson A., DeFouw E. R., LaBrot Z. C., Olmi D. J., Mong M. D. (In press). Welcome, learn, thrive: Enhancing class-wide prosocial behavior through positive greetings at the door. Journal of Positive Behavior Interventions.

[B66-behavsci-15-01640] LeBel T. J., Chafouleas S. M., Britner P. A., Simonsen B. (2013). Use of a daily report card in an intervention package involving home-school communication to reduce disruptive behavior in preschoolers. Journal of Positive Behavior Interventions.

[B67-behavsci-15-01640] LeGray M. W., Dufrene B. A., Mercer S., Olmi D. J., Sterling H. (2013). Differential reinforcement of alternative behavior in center-based classrooms: Evaluation of pre-teaching the alternative behavior. Journal of Behavioral Education.

[B68-behavsci-15-01640] Liming K. W., Grube W. A. (2018). Wellbeing outcomes for children exposed to multiple adverse experiences in early childhood: A systematic review. Child and Adolescent Social Work Journal.

[B69-behavsci-15-01640] Loomis A. M. (2018). The role of preschool as a point of intervention and prevention for trauma-exposed children: Recommendations for practice, policy, and research. Topics in Early Childhood Special Education.

[B70-behavsci-15-01640] Lyon A. R., Gershenson R. A., Farahmand F. K., Thaxter P. J., Behling S., Budd K. S. (2009). Effectiveness of Teacher-Child Interaction Training (TCIT) in a preschool setting. Behavior Modification.

[B71-behavsci-15-01640] Machado S. A., Anderson P. N. (2023). The perspectives of preschool teachers regarding their ability to respond to various crises in the childcare center. Journal of Early Childhood Research.

[B72-behavsci-15-01640] Maynard B. R., Farina A., Dell N. A., Kelly M. S. (2019). Effects of trauma-informed approaches in schools: A systematic review. Campbell Systematic Reviews.

[B73-behavsci-15-01640] McLaughlin K. A., Koenen K. C., Hill E. D., Petukhova M., Sampson N. A., Zaslavsky A. M., Kessler R. C. (2013). Trauma exposure and posttraumatic stress disorder in a national sample of adolescents. Journal of the American Academy of Child & Adolescent Psychiatry.

[B74-behavsci-15-01640] Merrick M. T. (2019). Vital signs: Estimated proportion of adult health problems attributable to adverse childhood experiences and implications for prevention—25 States, 2015–2017. MMWR Morbidity and Mortality Weekly Report.

[B75-behavsci-15-01640] Mullins C. A., Panlilio C. C. (2021). Exploring the mediating effect of academic engagement on math and reading achievement for students who have experienced maltreatment. Child Abuse & Neglect.

[B76-behavsci-15-01640] National TF-CBT Certification Program (2025). https://tfcbt.org/certification/.

[B77-behavsci-15-01640] Obradović J., Sulik M. J., Armstrong-Carter E. (2021). Taking a few deep breaths significantly reduces children’s physiological arousal in everyday settings: Results of a preregistered video intervention. Developmental Psychobiology.

[B78-behavsci-15-01640] Orengo-Aguayo R., Stewart R. W., de Arellano M. A., Suárez-Kindy J. L., Young J. (2019). Disaster exposure and mental health among Puerto Rican youths after Hurricane Maria. JAMA Network Open.

[B79-behavsci-15-01640] Panlilio C. C. (2019). Trauma-informed schools.

[B80-behavsci-15-01640] Panlilio C. C., Jones Harden B., Harring J. (2018). School readiness of maltreated preschoolers and later school achievement: The role of emotion regulation, language, and context. Child Abuse & Neglect.

[B81-behavsci-15-01640] Pasqua J. L., Dufrene B. A., LaBrot Z. C., Radley K., Dart E. H., Lown E. (2021). Evaluating the independent group contingency: “Mystery Student” on improving behaviors in head start classrooms. Psychology in the Schools.

[B82-behavsci-15-01640] Pechtel P., Pizzagalli D. A. (2011). Effects of early life stress on cognitive and affective function: An integrated review of human literature. Psychopharmacology.

[B83-behavsci-15-01640] Perrigo J. L., Palmer Molina A., Hurlburt M. S., Finno-Velasquez M. (2024). Exploring the drivers of child maltreatment under-and overreporting: A qualitative study. Families in Society.

[B84-behavsci-15-01640] Pokorski E. A., Barton E. E., Ledford J. R. (2017). A review of the use of group contingencies in preschool settings. Topics in Early Childhood Special Education.

[B85-behavsci-15-01640] Rajaraman A., Hanley G. P., Gover H. C., Staubitz J. L., Staubitz J. E., Simcoe K. M., Metras R. (2022). Minimizing escalation by treating dangerous problem behavior within an enhanced choice model. Behavior Analysis in Practice.

[B86-behavsci-15-01640] Riggs L., Landrum T. (2023). Trauma-informed PBIS: How educators can combine evidence-based practices for behavior management with trauma-informed care. Beyond Behavior.

[B87-behavsci-15-01640] Royer D. J., Lane K. L., Dunlap K. D., Ennis R. P. (2019). A systematic review of teacher-delivered behavior-specific praise on K-12 student performance. Remedial and Special Education.

[B88-behavsci-15-01640] Ruzek J. I., Brymer M. J., Jacobs A. K., Layne C. M., Vernberg E. M., Watson P. J. (2007). Psychological first aid. Journal of Mental Health Counseling.

[B89-behavsci-15-01640] Sachser C., Berliner L., Holt T., Jensen T. K., Jungbluth N., Risch E., Rosner R., Goldbeck L. (2017). International development and psychometric properties of the Child and Adolescent Trauma Screen (CATS). Journal of Affective Disorders.

[B90-behavsci-15-01640] Sacks V., Murphey D. (2018). The prevalence of adverse childhood experiences, nationally, by state, and by race or ethnicity. Child Trends.

[B92-behavsci-15-01640] Saunders B. E., Adams Z. W. (2014). Epidemiology of traumatic experiences in childhood. Child and Adolescent Psychiatric Clinics of North America.

[B93-behavsci-15-01640] Schatz J. N., Smith L. E., Borkowski J. G., Whitman T. L., Keogh D. A. (2008). Maltreatment risk, self-regulation, and maladjustment in at-risk children. Child Abuse & Neglect.

[B94-behavsci-15-01640] Schultz D., Tharp-Taylor S., Haviland A., Jaycox L. (2009). The relationship between protective factors and outcomes for children investigated for maltreatment. Child Abuse & Neglect.

[B95-behavsci-15-01640] Shepley C., Grisham-Brown J. (2019). Multi-tiered systems of support for preschool-aged children: A review and meta-analysis. Early Childhood Research Quarterly.

[B96-behavsci-15-01640] Skar A. M. S., Jensen T. K., Harpviken A. N. (2021). Who reports what? A comparison of child and caregivers reports of child trauma exposure and associations to post-traumatic stress symptoms and functional impairment in child and adolescent mental health clinics. Research on Child and Adolescent Psychopathology.

[B97-behavsci-15-01640] Smith S. D., Rivera F. A. P., DeFouw E. R., Walbridge F., Harris T., Wilde Z. C., Cotter M., Reichow B. (2025). The good behavior game as a universal preventive intervention: A systematic review of its long-term effects. Prevention Science.

[B98-behavsci-15-01640] Snell M. E., Berlin R. A., Voorhees M. D., Stanton-Chapman T. L., Hadden S. (2012). A survey of preschool staff concerning problem behavior and its prevention in Head Start classrooms. Journal of Positive Behavior Interventions.

[B99-behavsci-15-01640] Spence R., Kagan L., Kljakovic M., Bifulco A. (2021). Understanding trauma in children and young people in the school setting. Educational and Child Psychology.

[B100-behavsci-15-01640] Stanton-Chapman T. L., Walker V., Jamison K. R. (2014). Building social competence in preschool: The effects of a social skills intervention targeting children enrolled in head start. Journal of Early Childhood Teacher Education.

[B101-behavsci-15-01640] Strand V. C., Sarmiento T. L., Pasquale L. E. (2005). Assessment and screening tools for trauma in children and adolescents: A review. Trauma, Violence, & Abuse.

[B91-behavsci-15-01640] Substance Abuse and Mental Health Services Administration (2014). SAMHSA’s concept of trauma and guidance for a trauma-informed approach.

[B102-behavsci-15-01640] Sugai G., Horner R. R. (2006). A promising approach for expanding and sustaining school-wide positive behavior support. School Psychology Review.

[B103-behavsci-15-01640] Sun Y., Blewitt C., Minson V., Bajayo R., Cameron L., Skouteris H. (2024a). Trauma-informed interventions in early childhood education and care settings: A scoping review. Trauma, Violence & Abuse.

[B104-behavsci-15-01640] Sun Y., Tamblyn A., Morris H., Boothby C., Skouteris H., Blewitt C. (2024b). Early childhood and primary school teachers’ experiences and needs in working with trauma-impacted children: A systematic review and thematic synthesis. Children and Youth Services Review.

[B105-behavsci-15-01640] The National Child Traumatic Stress Network (2025). Effects. https://www.nctsn.org/what-is-child-trauma/trauma-types/early-childhood-trauma/effects.

[B106-behavsci-15-01640] Thielemann J. F. B., Kasparik B., König J., Unterhitzenberger J., Rosner R. (2022). A systematic review and meta-analysis of trauma-focused cognitive behavioral therapy for children and adolescents. Child Abuse & Neglect.

[B107-behavsci-15-01640] Thomas M. S., Crosby S., Vanderhaar J. (2019). Trauma-informed practices in schools across two decades: An interdisciplinary review of research. Review of Research in Education.

[B108-behavsci-15-01640] Tobin M., Oldfield J. (2016). Childhood trauma: Developmental pathways and implications for the classroom.

[B109-behavsci-15-01640] Vachhani S. S., Riley H. O., Miller A. L., Ellis J. M., Herrenkohl T. I. (2025). Trauma-Informed Programs and Practices for Schools (TIPPS): A promising model of system change to lessen pandemic effects on schools. International Journal of School & Educational Psychology.

[B110-behavsci-15-01640] Villalobos B. T., Dueweke A. R., Orengo-Aguayo R., Stewart R. W. (2023). Patient perceptions of trauma-focused telemental health services using the Telehealth Satisfaction Questionnaire (TSQ). Psychological Services.

[B111-behavsci-15-01640] von Schulz J. H., Dufrene B. A., LaBrot Z. C., Tingstrom D. H., Olmi D. J., Radley K., Mitchell R., Maldonado A. (2018). An evaluation of the relative effectiveness of function-based consequent and antecedent interventions in a preschool setting. Journal of Applied School Psychology.

[B112-behavsci-15-01640] Wade D., Crompton D., Howard A., Stevens N., Metcalf O., Brymer M., Ruzek J., Watson P., Bryant R., Forbes D. (2014). Skills for Psychological Recovery: Evaluation of a post-disaster mental health training program. Disaster Health.

[B113-behavsci-15-01640] Wang L., Norman I., Xiao T., Li Y., Leamy M. (2021). Psychological first aid training: A scoping review of its application, outcomes and implementation. International Journal of Environmental Research and Public Health.

[B114-behavsci-15-01640] Wang X., Heath R. D., Majewski D., Blake C. (2022). Adverse childhood experiences and child behavioral health trajectory from early childhood to adolescence: A latent class analysis. Child Abuse & Neglect.

[B115-behavsci-15-01640] Wassink-de Stigter R., Kooijmans R., Asselman M. W., Offerman E. C. P., Nelen W., Helmond P. (2022). Facilitators and barriers in the implementation of trauma-informed approaches in schools: A scoping review. School Mental Health.

[B116-behavsci-15-01640] Wiest-Stevenson C., Lee C. (2016). Trauma-informed schools. Journal of Evidence-Informed Social Work.

[B117-behavsci-15-01640] Wilcoxon L. A., Meiser-Stedman R., Burgess A. (2021). Post-traumatic stress disorder in parents following their child’s single-event trauma: A meta-analysis of prevalence rates and risk factor correlates. Clinical Child and Family Psychology Review.

[B118-behavsci-15-01640] Yoder M. L., Williford A. P. (2019). Teacher perception of preschool disruptive behavior: Prevalence and contributing factors. Early Education and Development.

